# Preovulatory Aging *In Vivo* and *In Vitro* Affects Maturation Rates, Abundance of Selected Proteins, Histone Methylation Pattern and Spindle Integrity in Murine Oocytes

**DOI:** 10.1371/journal.pone.0162722

**Published:** 2016-09-09

**Authors:** Hannah Demond, Tom Trapphoff, Deborah Dankert, Martyna Heiligentag, Ruth Grümmer, Bernhard Horsthemke, Ursula Eichenlaub-Ritter

**Affiliations:** 1 Institute of Human Genetics, University Hospital Essen, University Duisburg-Essen, Essen, Germany; 2 Institute of Gene Technology/Microbiology, University of Bielefeld, Bielefeld, Germany; 3 Institute of Anatomy, University Hospital Essen, University Duisburg-Essen, Essen, Germany; China Agricultural University, CHINA

## Abstract

Delayed ovulation and delayed fertilization can lead to reduced developmental competence of the oocyte. In contrast to the consequences of postovulatory aging of the oocyte, hardly anything is known about the molecular processes occurring during oocyte maturation if ovulation is delayed (preovulatory aging). We investigated several aspects of oocyte maturation in two models of preovulatory aging: an *in vitro* follicle culture and an *in vivo* mouse model in which ovulation was postponed using the GnRH antagonist cetrorelix. Both models showed significantly reduced oocyte maturation rates after aging. Furthermore, *in vitro* preovulatory aging deregulated the protein abundance of the maternal effect genes *Smarca4* and *Nlrp5*, decreased the levels of histone H3K9 trimethylation and caused major deterioration of chromosome alignment and spindle conformation. Protein abundance of YBX2, an important regulator of mRNA stability, storage and recruitment in the oocyte, was not affected by *in vitro* aging. In contrast, *in vivo* preovulatory aging led to reduction in *Ybx2* transcript and YBX2 protein abundance. Taken together, preovulatory aging seems to affect various processes in the oocyte, which could explain the low maturation rates and the previously described failures in fertilization and embryonic development.

## Introduction

Delayed ovulation leads to preovulatory aging of oocytes and "oocyte overripeness." It can occur during the entire reproductive life span in women in association with menstrual cycle irregularities [1]. Even though preovulatory aging is known to reduce oocyte quality and can cause developmental defects in the embryo in many different animal models, such as frogs, fish, urodeles, guinea pigs and rats [1], little is known about the underlying molecular mechanisms. Recently, an *in vivo* mouse model was established to investigate preovulatory aging in more detail [2]. In this model, ovulation was postponed by application of the gonadotropin releasing hormone (GnRH) antagonist cetrorelix, resulting in decreased embryo weight and increased embryo resorption [2]. Preovulatory aging may also occur during *in vitro* oocyte growth and maturation in follicle cultures. This system is becoming increasingly important in the use of cryopreservation or as an experimental method to assess influences of hormonal signaling, growth factors and toxic exposures on folliculogenesis, oocyte quality and developmental competence [3–10].

Using both the *in vivo* and *in vitro* model for preovulatory aging, we previously showed that transcript levels and poly(A) tail length of selected maternal effect genes (MEGs) like *Smarca4* and *Nlrp5* are altered by oocyte overripeness [11]. MEGs are expressed in the oocyte, but encode proteins that affect the phenotype of the embryo prior to and during the oocyte-to-embryo transition [12–14]. For example, *Smarca4* (also known as *Brg1*) encodes the catalytic subunit of the SWI/SNF-related complex that is required for chromatin remodeling during zygotic genome activation [15]. *Nlrp5* (also known as *Mater*) is part of the subcortical maternal complex (SCMC), a conserved subcortical domain in oocytes and zygotes that appears to harbor proteins involved in embryonic development and that has also been detected in human oocytes and embryos [16–18]. It has only recently been shown that NLRP5 protein is involved in mitochondrial activation, endoplasmic reticulum localization and calcium homeostasis in oocytes and early embryos [19–22]. It is currently unknown whether altered transcript levels of MEGs seen in preovulatory aging also affect the corresponding protein levels.

During oocyte growth, transcripts of the maternal effect genes, as well as other mRNAs, accumulate and are stored in the oocyte, due to the drop in transcription at the onset of oocyte maturation to low or even undetectable levels [23,24]. To ensure stability of the transcripts and their poly(A) tail after this transcriptional silencing, the germ-cell specific RNA-binding protein YBX2 (also known as MSY2) is required [25–27]. YBX2 is one of the most abundant proteins in the growing oocyte with vital functions [26]. Loss of YBX2 in the oocyte causes mRNA instability and deterioration of transcriptional quiescence leading to major deregulation of the transcriptome, which ultimately impairs oocyte maturation and decreases fertilization rates in mice [27,28].

Apart from regulation of expression at the transcript and proteome level, epigenetic regulations, such as histone modifications, are known to occur prior to and during maturation and are relevant for gene expression and particularly chromosome integrity that influence chromosome segregation in oocytes [29,30]. Trimethylation of histone 3 lysine 9 (H3K9me3) has been associated with heterochromatin formation and gene silencing, pericentromeres in oocytes, and chromosome stability during meiosis [31–38]. Conditional deletion of the H3K9 methyltransferase Setdb1 leads to meiotic arrest, disruption of chromatin condensation and spindle dynamics and altered transcript abundance, resulting in lower oocyte maturation rates and impaired embryonic development [37,38].

In the present study, we used the *in vitro* and *in vivo* models for preovulatory aging in the mouse to investigate the effects of oocyte overripeness on oocyte maturation and protein expression of selected maternal effect genes and YBX2. Furthermore, we analyzed the histone modification H3K9me3 and assessed chromosome stability to gain deeper insight into the processes during oocyte ripening and their temporal regulation.

## Materials and Methods

### Ethics Statement

The study was conducted in compliance with the Guide for the Care and Use of Laboratory Animals of the German Government. The protocol was approved by the Committee of Ethics of Animal Experiments (Landesamt für Natur, Umwelt und Verbraucherschutz, LANUV AZ 84–02.04.2011.A374). All animals (see below) were kept under standard conditions (12 h dark and 12 h light cycle, food and water *ad libitum*) in the Central Animal Laboratory of the University Hospital Essen and Bielefeld University Animal House. Animals were acquired from the breeding colonies of the animal facilities.

### In vivo preovulatory aging of oocytes

Preovulatory aging was defined as oocyte overripeness due to prolonged growth of oocytes before ovulation induction. To achieve prolonged follicle growth and oogenesis *in vivo*, ovulation was delayed in superovulated 4-6 week old C57Bl/6J female mice by application of the GnRH antagonist cetrorelix (Cetrotide, Merck-Serono), as described previously [2,11]. In short, mice were stimulated by intraperitoneal injection of 10 IU pregnant mare serum gonadotropin (PMSG; Intergonan, MSD) to induce follicle growth. In addition, 50 μg of cetrorelix was applied subcutaneously daily to block endogenous triggering of ovulation. Control oocytes were obtained after ovulation induction by 10 IU human chorionic gonadotropin (hCG; Ovogest, MSD) 48 h after PMSG treatment. Preovulatory oocyte aging (PreOA) was achieved by prolonging cetrorelix-treatment for an additional 4 days while maintaining stimulation of follicle growth with 10 IU PMSG every second day. Mice were anesthetized with isoflurane and sacrificed by cervical dislocation 14-16 h after hCG application for oocyte collection from the oviduct. Cumulus cells were removed from oocytes by brief enzymatic treatment with hyaluronidase (10 mg/ml, Sigma-Aldrich). Oocytes that were used for immunohistochemical analysis were immediately processed according to the protocols below. Oocytes that were analyzed by qRT-PCR were stored at -80°C until further usage. The number of retrieved oocytes from each mouse was counted and the percentage of degenerated oocytes was calculated. Concomitantly, to evaluate the possible effect of the GnRH antagonist on oocyte maturation, another control group not receiving cetrorelix was analyzed.

### In vitro preovulatory aging of oocytes

Preantral follicles were isolated after mechanical release from the ovaries of 12 to 14 d old female F1 hybrid C57Bl/6J x CBA/Ca mice. Follicle culture was performed as previously described [9,11]. Briefly, preantral follicles with a diameter of 110-130 μm were cultured individually at 5% CO_2_ and 37°C in αMEM Glutamax (Invitrogen) for 12 d (control) or 16 d. Twelve days of culture time corresponds to the time required for *in vitro* growth and maturation of oocytes and is used in standard protocols [3,6,9]. Culture medium was supplemented with 10 mIU/ml recombinant follicle stimulating hormone (rFSH; Gonal-f; Merck-Serono), 5 μg/ml insulin, 5 μg/ml transferrin, 5 ng/ml sodium selenite (Insulin, Transferrin, Sodium Selenite, ITS, Sigma-Aldrich), and 5% fetal calf serum (Invitrogen) covered with mineral oil (Invitrogen). Medium with rFSH was replenished every fourth day. Recombinant luteinizing hormone (rLH; Luveris; 10 mIU/ml; Merck-Serono) was added once at the beginning of the *in vitro* follicle culture.

Resumption of maturation was induced on day 12 (control) or day 16 (PreOA) of culture by 5 ng/ml recombinant epidermal growth factor (rEGF, Promega) and 1.5 IU/ml rhCG (Ovitrelle; kindly donated by Merck-Serono). MII oocytes were retrieved 18 h post rhCG/rEGF induction. Cumulus cells were removed by brief hyaluronidase treatment. MII oocytes were further processed for immunostaining or stored at -80°C for transcript analysis. Oocyte maturation was analyzed by determining the percentage of follicles that developed to the MII stage, arrested at the stage of germinal vesicle breakdown (GVBD) or at the germinal vesicle (GV) stage, or were degenerated at the end of culture (control: day 13, PreOA: day 17).

### Semi-quantitative assessment of SMARCA4 and NLRP5 protein

Protein abundance of SMARCA4 and NLRP5 was assessed after preovulatory aging *in vitro* for 4 d at GV stage. Cumulus-free oocytes were fixed for 30 min at 4°C (4% paraformaldehyde in PBS), permeabilized for 15 min at RT (0.5% Triton X-100 in PBS) and blocked for 1 h at 37°C (0.1% w/v BSA and 0.1% v/v Tween20 in PBS). GVs were incubated for 1 h at RT in rabbit polyclonal anti-SMARCA4 or rabbit polyclonal anti-NLRP5 antibody (both Santa Cruz; sc-10768 and sc-134842). After three washing steps (0.1% v/v Tween20 in PBS) at RT for 15 min each, cells were incubated in anti-rabbit TRITC (Sigma-Aldrich) for 1 h at RT. Chromosomes were counterstained with Sytox Green dye (Invitrogen) in parallel with SMARCA4 staining or with 4,6-diamindino-2-phenylindole (DAPI; Sigma-Aldrich) together with NLRP5 staining. The semi-quantitative protein abundance was determined for the whole oocyte by assessing relative fluorescence intensity compared to controls as mean value in arbitrary units [a.u.±SEM] after normalization against background fluorescence using a Leica LCSSP2 confocal laser scanning microscope and the Leica Lite software.

### Semi-quantitative assessment of YBX2 protein

Protein level and localization of YBX2 was assessed in controls or after preovulatory aging *in vitro* at GV and MII stage and after preovulatory aging *in vivo* at MII stage. Oocytes were fixed for 60 min at RT (4% w/v paraformaldehyde in PBS), permeabilized for 15 min at RT (0.1% v/v Triton X100 in PBS) and blocked for 30 min at RT (0.1% w/v BSA and 0.01% v/v Tween20 in PBS). Cells were incubated in goat polyclonal anti-YBX2 (Santa Cruz; sc-21316) for 1 h at RT and then washed 3 times (0.1% w/v BSA and 0.01% v/v Tween20 in PBS) at RT for 15 min each. This was followed by incubation in anti-goat Cy3 (Sigma-Aldrich) overnight at 4°C. Chromosomes were counterstained with DAPI. Quantitative fluorescence intensity [a.u.±SEM] of aged oocytes was normalized against background fluorescence and compared to normalized staining intensity of controls using Leica LCSSP2 confocal laser scanning microscope and Leica Lite software.

### Analysis of histone H3K9 trimethylation

Trimethylation of histone H3 lysine K9 (H3K9me3) was analyzed in *in vivo* and *in vitro-grown* preovulatory-aged MII oocytes. Immunostaining and assessment of signal intensity was done as previously described [10]. In brief, the zona pellucida was removed from MII oocytes after brief pronase digestion, followed by fixation (4% w/v paraformaldehyde in PBS), permeabilization (0.5% v/v Triton X100) and blocking steps (0.2% w/v sodium azide, 2% v/v normal goat serum, 0.2% w/v powdered milk, 0.1 M v/v glycine, 0.01% v/v Triton X100 and 1% w/v BSA in PBS). Immunostaining was conducted with a rabbit polyclonal anti-trimethylated H3K9 (Epigentek; A-4036-025_EP) and a TRITC-conjugated secondary anti-rabbit antibody (Sigma-Aldrich) followed by counterstaining of DNA with DAPI. Images of H3K9me3 staining were recorded with a Zeiss Axiophot fluorescence microscope with set exposure time. Relative fluorescence intensity [a.u.±SEM] of aged oocytes in comparison to controls was quantified with the ImageJ software after normalization against background staining.

### Chromatin status, spindle conformation and chromosomal alignment

Chromatin status at GV stage (as an indicator for transcriptional activity and developmental competence) was analyzed in fixed and DAPI-stained *in vitro-grown* oocytes at day 12 or 16 as described previously [9].

Meiotic spindle and chromosomal alignment were analyzed after preovulatory aging in MII oocytes grown *in vitro*. Oocytes were fixed (2% w/v paraformaldehyde, 0.1% v/v Triton X100, 1 μM taxol, 0.01% w/v aprotinine, 0.1 M Pipes, pH 6.9, 5 mM MgCl_2_ and 2.5 mM EGTA) for 45 min at 37°C and blocked (0.2% w/v sodium azide, 2% v/v normal goat serum, 0.2% w/v powdered milk, 0.1 M v/v glycine, 0.01% v/v Triton X100 and 1% w/v BSA in PBS) for 60 min at 37°C. For immunostaining monoclonal mouse anti-α-tubulin antibody and FITC-conjugated secondary anti-mouse antibody (both Sigma-Aldrich) were used. Counterstaining of chromosomes was conducted with DAPI. Spindle morphology and chromosome alignment were analyzed by z-axial scanning with sequential scan mode using a Leica LCSSP2 confocal laser scanning microscope. A spindle abnormality was defined as a spindle showing at least one of the following criteria: 1) Spindle is not bipolar, 2) asymmetric or 3) very small. Chromosomes alignment was assessed using the following 3 categories: 1) aligned, 2) scattered, 3) displaced.

### RNA isolation

Total RNA was obtained from 3 pools of 20 MII oocytes for both controls and preovulatory-aged oocytes. For RNA preparation the Arcturus PicoPure RNA Isolation Kit (Life Technologies) was used according to manufacturer’s instructions with small adaptations: After incubation of oocytes in extraction buffer for 30 min at 42°C, the cell extract was homogenized on a QiaShredder Column (Qiagen) for 5 min at full speed. RNA was depleted from any DNA by DNase I digestion (Qiagen) according to the manufacturer’s protocol.

### qRT-PCR analysis

Transcript levels of *Ybx2* were determined in control and preovulatory-aged MII oocytes by qRT-PCR analysis. For reverse transcription of total RNA from pooled oocytes, MuLV transcriptase (Life Technologies) was employed according to manufacturer’s instructions using random hexamer primers (Life Technologies). Quantitative RT-PCR was performed on the Light Cycler 480II (Roche) using the Universal Probe Library (Roche) and primers obtained from Biomers (*Ybx2*: F 5’-ttctgcggagtgttggagat-3’, R 5’-aggcccagtgacattagcag-3’). Samples were incubated for 10 min at 95°C and then amplified in 50 cycles of 10 s at 95°C, 30 s at 60°C and 1 s at 72°C. Each sample was measured in triplicate. Since expression level of housekeeping genes is known to be affected by oocyte *in vitro* maturation [39] relative quantification of gene expression was analyzed by calculation of the fold change of aged oocytes in comparison to controls using a standard curve of Luciferase control RNA (Promega).

### Statistics

Jarque-Bera or Shapiro-Wilk pre-tests were employed to assess non- or parametric characteristics of each sample cohort. For the non-parametric cohorts of the number of oocytes after ovulation *in vivo*, H3K9me3 analysis, *Ybx2* mRNA levels, and semi-quantitative protein abundance of SMARCA4, NLRP5 and YBX2, the Mann-Whitney U-test post-test was used. Oocyte maturation rate, chromatin status, spindle integrity and chromosomal alignment of *in vitro*-grown oocytes were analyzed using the Chi^2^-test. All results are indicated as Mean ± SEM. Differences were regarded as significant in case of *p* < 0.05.

## Results

### Oocyte maturation rates decline after in vitro and in vivo preovulatory aging

**Fig 1:**
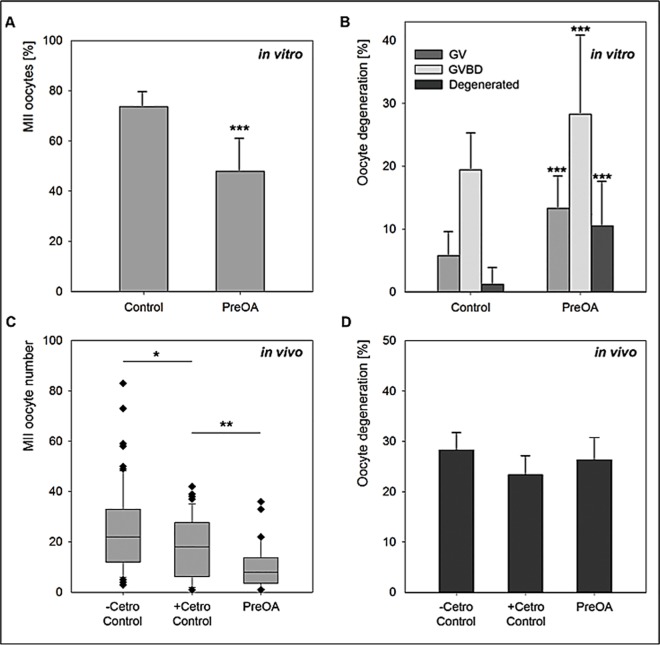
**Oocyte maturation after *in vitro* (A,B) and *in vivo* (C,D) preovulatory aging (PreOA). A)** Decrease in MII oocytes after PreOA in follicle culture (n = 572) compared to control (n = 721). **B)** Increased percentage of oocytes arrested at GV (Control: *n* = 33, PreOA: *n* = 66) and GVBD stage (Control: *n* = 111, PreOA: *n* = 204) or degenerated (Control: *n* = 7, PreOA: *n* = 76) in preovulatory-aged *in vitro*-cultured oocytes compared to controls. **C)** Decrease in mean number of ovulated MII oocytes retrieved per mouse in control mice receiving cetrorelix (+Cetro Control; *n* = 38) compared to controls not receiving cetrorelix (-Cetro Control; *n* = 38). Preovulatory aging (*n* = 32) further reduced the oocyte number compared to the +Cetro Control group. **D)** Percentage of degenerated oocytes showed no significant differences between the–Cetro Control, +Cetro Control and PreOA group. Significant difference to controls: * *p* < 0.05, ** *p <* 0.01, *** *p <* 0.001.

To investigate the effect of preovulatory aging *in vitro* on oocyte maturation, the percentage of oocytes that matured to the MII stage in preantral follicle culture was calculated. In total, 572 control and 721 preovulatory-aged (PreOA) follicles were analyzed. While oocytes in 73.6% of control follicles successfully reached the MII stage, only 47.9% of the preovulatory-aged oocytes matured to MII (*p* < 0.001; Fig 1A). Preovulatory aging caused a highly significant increase in the percentage of oocytes that arrested at the germinal vesicle stage (GV; 5.8% in control vs. 13.3% in PreOA; *p* < 0.001) or became arrested after germinal vesicle breakdown (GVBD; 19.4% in control vs. 28.3% in PreOA; *p* < 0.001; Fig 1B). Furthermore, oocyte degeneration rate increased significantly from 1.2% in control follicles to 10.5% in the preovulatory-aged group (*p* < 0.001; Fig 1B).

To analyze the effect of preovulatory aging on the number of oocytes that ovulated per mouse *in vivo*, ovulation was postponed by application of the GnRH antagonist cetrorelix. To determine whether cetrorelix *per se* has an effect on oocyte maturation, control mice which received cetrorelix (+Cetro) as well as control mice not receiving cetrorelix (-Cetro) were investigated. For each of the two control groups (+Cetro and -Cetro), 38 mice were analyzed. Cetrorelix application in control mice significantly reduced the mean number of ovulated MII oocytes recovered per mouse from 22.2 ± 3.19 to 19.5 ± 2.00 oocytes (*p* < 0.05; Fig 1C). To exclude that the observed effects were due to cetrorelix application rather than follicular overmaturity, control +Cetro mice were used for all further experiments as controls.

After 4 days of *in vivo* preovulatory aging, an average of 9.9 ± 1.71 oocytes (*n* = 32) was retrieved per mouse, which was significantly less than in the +Cetro control group (*p* < 0.01; Fig 1C). To investigate whether this reduced number of oocytes also correlates with oocyte quality, as observed in the *in vitro-*aged groups, we determined the rate of oocyte degeneration among ovulated oocytes per mouse. We did not see significant differences between the numbers of *in vivo* ovulated oocytes that exhibited degeneration between any of the 3 *in vivo* groups analyzed (Fig 1D). We assume that degenerated oocytes were incapable to become ovulated, but the possible meiotic arrest and retention of oocytes with GVBD or GV stage in ovary was not evaluated. Taken together, preovulatory aging *in vitro* and *in vivo* led to a significant impairment of oocyte maturation and ovulation rate.

### Preovulatory oocyte aging affects protein abundance of selected maternal effect genes and YBX2

**Fig 2:**
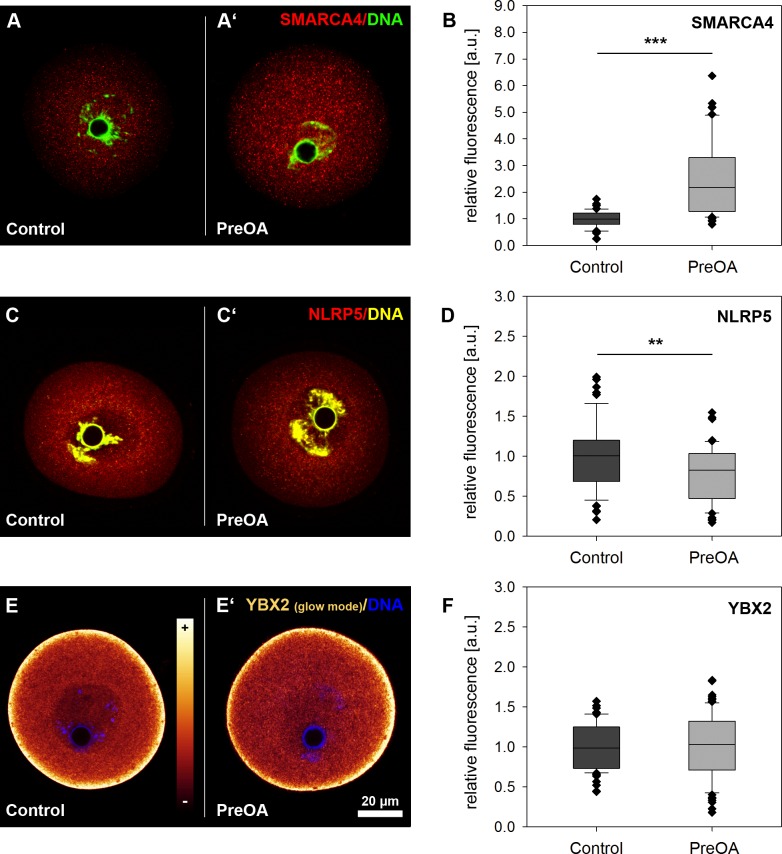
**Relative abundance of the maternal effect proteins SMARCA4 (A, B), NLRP5 (C, D) and YBX2 (E, F) in control and *in vitro* preovulatory-aged (PreOA) GV oocytes. A, A’, C, C’**, **E** and **E’)** Representative images of oocytes stained with antibodies against SMARCA4, NLRP5 and YBX2. YBX2 staining **(E, E’**) is shown in images of oocytes examined by a glow mode by confocal laser scanning microscopy. The coloring of the scale in **E** corresponds to relative expression levels. **B, D, F**) Box plot analysis of relative abundance of SMARCA4 (**B**), NLRP5 (**D**) and YBX2 (**F**) proteins in PreOA oocytes compared to controls. Scale bar in **E’** = 20 μm and also applies to **A**, **A’**, **C**, **C’** and **E**. Significant difference to control: ** *p <* 0.01, *** *p <* 0.001.

Transcripts and proteins of MEGs accumulate during oocyte growth and are required to guide the germ cell through the processes of fertilization and oocyte-to-embryo transition [14]. Storage and timely recruitment of some of these MEGs relies on proteins like YBX2 and the stage-specific regulation of mRNA translation [26,27,40]. As there was previous evidence for changes in abundance of mRNAs of MEGs at MII stage in preovulatory-aged oocytes [11], we assessed protein abundance after *in vitro* aging in the *in vitro* follicle culture model. Immunohistochemical analysis was used to localize and quantify the abundance of two maternal effect proteins, SMARCA4 and NLRP5, and the RNA-binding protein YBX2 in GV oocytes from *in vitro* follicle culture. SMARCA4 protein was located in both the nucleus and the cytoplasm of *in vitro-grown* control GV oocytes (Fig 2A). The presence of SMARCA4 in the cytoplasm was unexpected, but may be due to its role as a maternal effect protein with a function in the early embryo. Analysis of relative protein abundance using semiquantitative confocal microscopy of oocytes showed a similar distribution but significantly increased protein abundance of SMARCA4 protein in the PreOA group compared to controls (Fig 2A’ and 2B; Control: *n* = 63, PreOA: *n* = 68; *p* < 0.001).

NLRP5 protein was predominantly located in the cytoplasm of control GV oocytes (Control: *n* = 58; Fig 2C). In contrast to SMARCA4, the relative protein abundance of NLRP5 decreased slightly but significantly compared to controls after preovulatory aging *in vitro*, while protein distribution was not visibly affected (Fig 2C’ and 2D; PreOA: *n* = 55; *p* < 0.01).

The germ cell specific RNA-binding protein YBX2 is essential for stage specific regulation of the transcriptome. Immunohistochemical staining of the YBX2 protein in GV controls from *in vitro* follicle culture was strongly enriched in the subcortical ribonucleoprotein (RNP) domain and accumulated in the cytosol, but was less prominent in the nucleus (Fig 2E, Control: *n =* 60), as described previously by Yu and colleagues [25]. *In vitro* preovulatory-aged oocytes did not significantly affect YBX2 protein abundance or localization at GV stage (Fig 2E’ and 2F; PreOA: *n =* 74).

**Fig 3:**
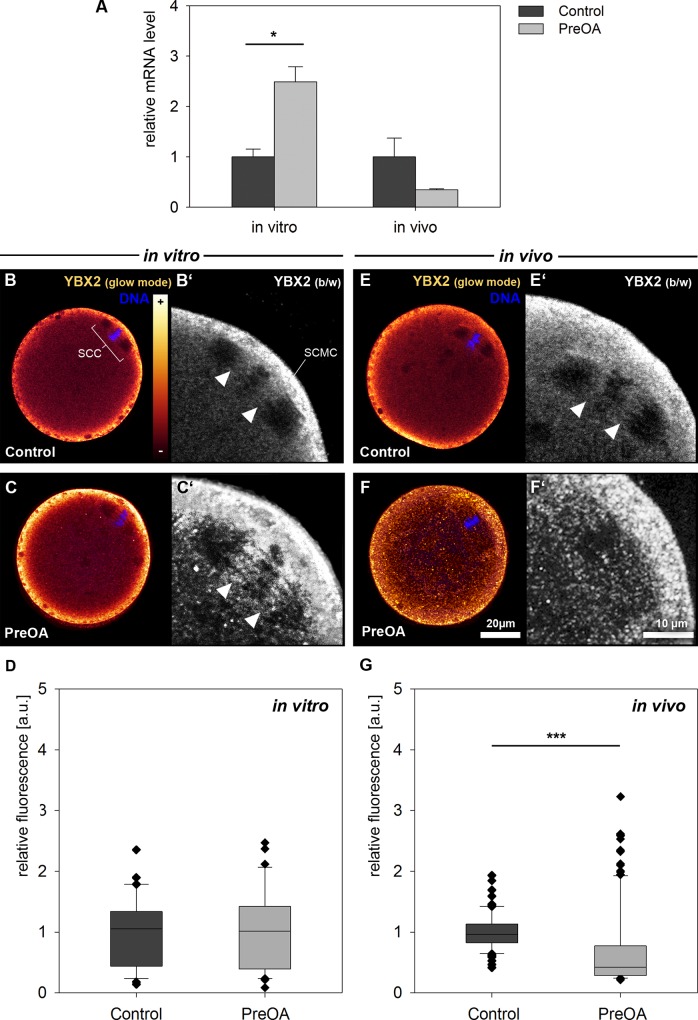
***Ybx2* mRNA levels (A) and YBX2 protein localization and abundance in control and preovulatory-aged (PreOA) MII oocytes grown *in vitro* (B-D) or *in vivo* (E-G).** SCC: Spindle chromosome complex; SCMC: Subcortical maternal complex. Arrowheads in **B**’, **C’**, and **E’**: spindle domain right and left of chromosomes that is enriched for YBX2. Scale bar in **F** = 20 μm and also applies to **B, C** and **E**. Scale bar in **F**’ = 10 μm and applies to **B’, C’** and **E’**. Significant difference to control: * *p* < 0.05, *** *p <* 0.001.

We had previously studied the abundance of mRNA of *Smarca4* and *Nlrp5* in preovulatory-aged MII oocytes in the same *in vitro* and *in vivo* model, but not that of *Ybx2* [11]. We therefore assessed transcript levels of *Ybx2* in MII oocytes that had aged 4 days *in vivo* or *in vitro*. Quantitative RT-PCR analysis revealed significantly increased transcript abundance of *Ybx2* mRNA in MII oocytes after *in vitro* preovulatory aging compared to controls in three biological replicates of 20 pooled oocytes each (Fig 3A; *p* < 0.05). In contrast, MII oocytes tended to have decreased *Ybx2* transcript levels compared to control oocytes after *in vivo* preovulatory aging (*n =* 3; *p =* 0.068).

**Fig 4:**
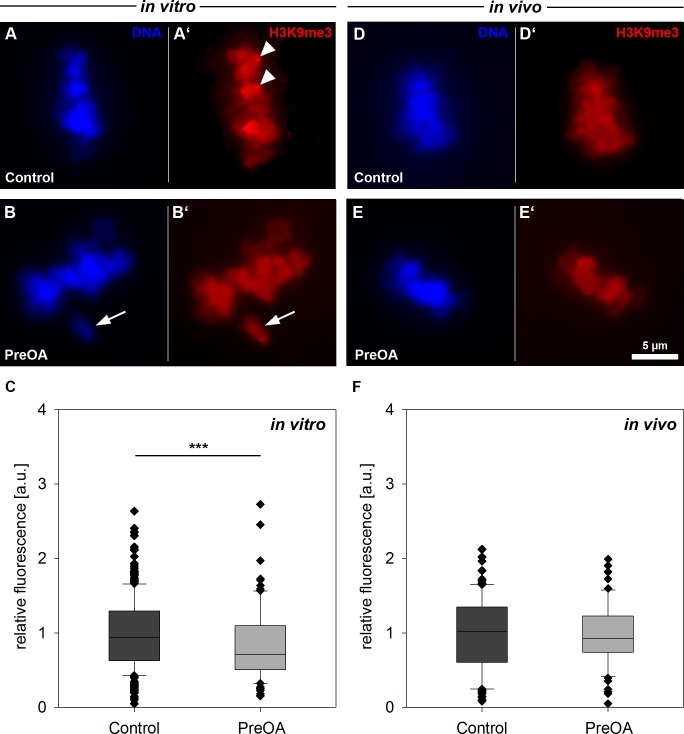
**Distribution and abundance of histone 3 lysine 9 trimethylation (H3K9me3) in control and preovulatory-aged (PreOA) MII oocytes grown *in vitro* (A-C) or *in vivo* (D-F).** Arrowheads in **A’** indicate pericentromeric heterochromatin enriched for H3K9me3. **A, B, D, E:** DAPI stained MII chromosomes. **A’, B’, D’, E**’: anti-H3K9me3 staining. Arrow in **B, B’**: Unaligned chromosome. Scale bar in in **E** = 5 μm and also applies to **A-E’**. Significant difference to control: *** *p <* 0.001.

Immunohistochemical staining of the YBX2 protein in control MII stage oocytes revealed an enrichment of this protein in the subcortical region and in the cytosol but also in the spindle chromosome complex (SCC) in controls of both the *in vitro* (*n =* 42) and *in vivo* groups (*n =* 83; Fig 3B and 3E). In the SSC, YBX2 was observed left and right proximal to the chromosomes, but not in the area of the more polar parts of the spindle (Fig 3B’ and 3E’). These results are consistent with a recent study of our group, indicating that YBX2 localizes in the SCC of *in vivo*-grown murine MII oocytes [41]. As for the *in vitro* preovulatory-aged MII stage, staining and quantification of relative fluorescence intensity showed no significant effect on protein localization or abundance compared to the control (Fig 3C and 3D). In contrast, in the *in vivo* preovulatory-aged MII oocytes there was a highly significant decrease in the overall protein abundance of the cortical region and the spindle chromosome complex (Figs 3F and 4G; Control: *n* = 41, PreOA: *n =* 120; *p <* 0.001). Although the overall distinct YBX2 pattern in the cytoplasm appeared similar to the control, particularly the staining in the SCC was diminished by aging (as shown in representative image in Fig 3F’).

Taken together, abundance of SMARCA4, NLRP5 and YBX2 protein in oocytes was affected differentially, pointing towards a fine-tuned, time- and stage-specific recruitment and processing of individual transcripts and proteins during the intrafollicular growth of oocytes. Aging appears to have a differential influence on the expression and/or storage of the investigated proteins.

### In vitro preovulatory aging decreases H3K9me3 in MII oocytes

We analyzed the epigenetic histone modification H3K9me3 that is proposed to be involved in gene silencing and stabilization of constitutive heterochromatin at pericentromeres during oocyte maturation [33,42]. Immunohistochemical staining for H3K9me3 was performed in both, the *in vitro*-ovulated MII oocytes from follicle culture and the *in vivo*-grown and ovulated MII oocytes. As expected, H3K9me3 was co-localized with chromosomes (Fig 4A and 4A’) and was enriched at pericentromeres (arrowheads in Fig 4A’), as previously described [10]. Relative H3K9me3 signal abundance was significantly reduced after *in vitro* preovulatory aging (Fig 4B, 4B' and 4C; PreOA: *n* = 91; *p* < 0.001) compared to controls (*n* = 266). *In vivo* preovulatory aging revealed a slight but not significant reduction of H3K9me3 abundance compared to the controls (Fig 4D–4F; Control: *n =* 106, PreOA: *n* = 65).

### Spindle integrity and chromosome alignment is impaired after preovulatory aging in vitro

**Fig 5:**
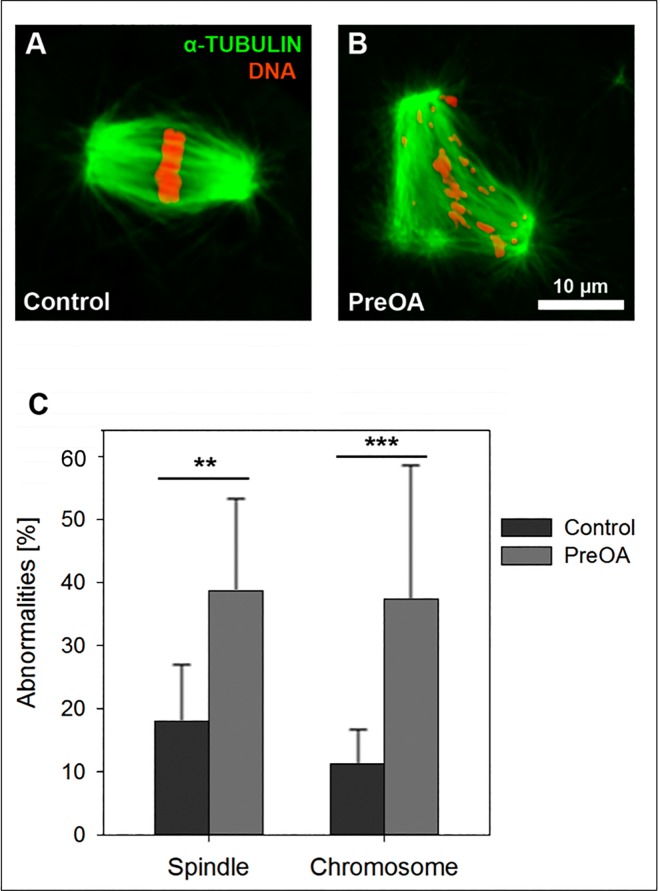
Spindle abnormalities and chromosome alignment in *in vitro* control and preovulatory-aged (PreOA) MII oocytes. Spindles (green) and chromosomes (red) in control **(A)** and PreOA MII oocytes **(B)** and the percentage of oocytes with spindle and chromosome abnormalities **(C)**. Scale bar in **B** = 10 μm and also applies to **A**. Significant differences between groups: ** *p <* 0.01; *** *p <* 0.001.

Since H3K9me3 is thought to be required for chromosomal stability and spindle functionality during meiosis [10,32] and was reduced in *in vitro* preovulatory-aged oocytes, as shown above, we analyzed spindle integrity and chromosome alignment in control MII oocytes and after *in vitro* preovulatory aging. In the majority of *in vitro-*grown control oocytes the spindle apparatus was intact and the chromosomes were well aligned at the metaphase plate (Fig 5A). Only 16 of 89 analyzed control oocytes (18.0%) showed slight spindle abnormalities and in 10 of 89 oocytes (11.2%) some chromosomes failed to align precisely at the equator. *In vitro* preovulatory aging induced a rise in spindle and chromosomal abnormalities (e.g. oocytes with three spindle poles and chromosomes scattered throughout the entire spindle; Fig 5B). In comparison to controls, the number of oocytes with spindle abnormalities (29 of 75 oocytes, 38.7%, *p <* 0.001) and number of oocytes with unaligned chromosomes (28 of 75 oocytes, 37.3%, *p <* 0.001) were significantly increased in the preovulatory-aged compared to the control group (Fig 5C).

## Discussion

The present study addressed the effects of delayed ovulation on several aspects of oocyte competence in two different models: a mouse *in vitro* follicle culture system and an *in vivo* mouse model. Oocyte maturation was severely affected by preovulatory aging in both the *in vitro* and *in vivo* systems. *In vitro* preovulatory aging led to impairments of protein abundance of the maternal effect genes *Smarca4* and *Nlrp5* as well as to aberrant H3K9me3, chromosome pattern failure and spindle abnormalities. After preovulatory aging *in vivo* the primary observation was that the germ cell factor YBX2 was reduced. These observations demonstrate that preovulatory aging disrupts regulation of various processes in the maturing oocyte which could explain the impairments previously described for embryo development after fertilization of preovulatory-aged oocytes [1,2].

We further observed an increased rate of maturation arrest and oocyte degeneration after *in vitro* preovulatory aging, which correlated with a decreased percentage of matured MII oocytes. Also *in vivo* aging caused a decline in the number of MII oocytes retrieved from the ampullae of females once ovulation was induced. Although there was no effect of aging on the numbers of degenerated, ovulated oocytes in the *in vivo* group, degenerating and arrested oocytes might become apoptotic in atretic follicles before ovulation thereby decreasing the ovulated oocyte yield.

Preovulatory aging *in vivo* may affect folliculogenesis, granulosa cell development and cumulus expansion due to an imbalanced hormonal homeostasis, since cetrorelix suppresses intrinsic gonadotropins. Decreased oocyte numbers of control mice receiving cetrorelix in comparison to controls without cetrorelix treatment is in accordance with findings in humans where treatment with cetrorelix is used to prevent a premature LH surge during ovarian stimulation. In humans, cetrorelix as well as other GnRH antagonists have been described to lower oocyte retrieval and fertilization rates in comparison to GnRH agonists [43,44]. In order to study the influence of intrafollicular aging rather than suppression of LH, all controls in the present study received cetrorelix but were stimulated to ovulate without delay. It is important to note that the effect of cetrorelix in normally cycling controls and the differences seen between the *in vitro* and *in vivo* model may be partially attributable to differences in regulating the LH and FSH secretion.

In a preceding study, we described a decline of *Nlrp5* transcript levels in MII mouse oocytes after *in vitro* preovulatory aging, while *Smarca4* transcript expression stayed stable [11]. Here, we now provide evidence that the decrease in *Nlrp5* mRNA correlates with a reduction of NLRP5 protein abundance in *in vitro* preovulatory-aged GV oocytes. *Nlrp5* transcript and NLRP5 protein levels have been shown to drop during oocyte maturation [27], suggesting that the expression of active protein is important for processes prior to ovulation, apart from putative functions in early embryogenesis. The precocious decrease of protein may therefore have adverse effects on oocyte maturation and developmental competence, and possibly during subsequent preimplantation embryogenesis. Loss of NLRP5 has also recently been shown to be associated with maternal aging and postovulatory aging in mice [41,45]. In postovulatory-aged oocytes the drop in NLRP5 protein also correlated with reduced H3K9me3 and chromosome misalignment [41], which is consistent with our observations in *in vitro* preovulatory aging. The direct link between aging and reduced NLRP5 levels remains to be analyzed in future studies. Nevertheless, the present study supports previous observations that this protein is particularly susceptible to temporal deregulation.

In contrast, SMARCA4 protein level increased in GV stage oocytes after *in vitro* preovulatory aging. Since SMARCA4 plays a role in chromatin remodeling after fertilization, the increase in protein in GV oocytes may affect the nucleosome landscape in the oocyte and/or early embryo. SMARCA4 is known to deacetylate H3K9 by interaction with histone deacetylase 1 (HDAC1) during preimplantation development, thereby allowing H3K9 methylation [46]. The precocious expression of SMARCA4 after preovulatory aging might therefore explain the changes observed in H3K9me3. Overall, it appears from the analysis of transcript and protein abundance that genes are differentially deregulated and that mRNA abundance is not directly predictive of protein abundance, supporting similar findings in postovulatory-aged MII oocytes [41]. It is likely that in oocytes, where maturation occurs in the absence of transcription, post-transcriptional regulation of protein synthesis and stability are especially important compared to other cell types [47]. Disturbance of these mechanisms can contribute to reduced oocyte quality and developmental capacity.

Since the YBX2 protein can affect poly(A) tail length, mRNA storage and translation, we investigated the expression of this protein in the oocyte. We did not observe a significant alteration in abundance and distribution of YBX2 protein at the GV or MII stage of development after *in vitro* preovulatory aging. However, decreased *Ybx2* mRNA and YBX2 protein levels were observed in MII oocytes after *in vivo* preovulatory aging, which might contribute to the deregulated abundance of transcript and poly(A) levels of several MEGs that were recently observed in *in vivo* preovulatory-aged MII oocytes [11]. The decrease in YBX2 protein levels observed only after *in vivo* preovulatory aging may be explained by a different environment in the ovary (e.g. in endocrine axis) compared to the *in vitro* culture system, leading to differences between *in vivo* and *in vitro*-aged oocytes as also previously seen at the transcript level [11].

YBX2 is phosphorylated by CDC2A (CDK1) during meiotic resumption, which releases transcripts bound to YBX2 making them accessible to translation machinery and susceptible to degradation during oocyte maturation [48]. Afterwards, YBX2 protein levels drop due to a decline in *Ybx2* mRNA during oocyte maturation [48]. The observed decrease in *Ybx2*/YBX2 transcript and protein abundance after *in vivo* preovulatory aging suggests a precocious recruitment of factors associated with YBX2 protein during aging. This hypothesis corresponds to a previous study showing early polyadenylation of maternal effect mRNAs in oocytes after *in vivo* preovulatory aging [11].

In addition to a perturbation in protein expression of MEGs, we observed a decrease in H3K9me3 after preovulatory aging in *in vitro*-grown oocytes. Histone posttranslational modifications appear particularly sensitive to aging of oocytes, not just in preovulatory aging, but also postovulatory aging and maternal aging [41,49,50]. Maternal aging can cause complete depletion of H3K9me3 in some oocytes [49]. We did not observe such dramatic effects during preovulatory aging. The decrease in H3K9me3 levels was more similar to that described for mouse postovulatory-aged oocytes [41]. H3K9me3 is required for genome stability, and histone methylation deficiency leads to chromosome disruption during meiosis [10,32,37,38]. In postovulatory-aged oocytes, we saw an increase in chromosome misalignments and spindle aberrations in parallel to a decrease in H3K9me3 [41]. Here, we also observed decreasing H3K9me3 levels concurrent with an increase in chromosome and spindle perturbations in *in vitro* preovulatory-aged oocytes. Such chromosomal aberrations could explain the increased rate of developmentally arrested oocytes in the follicle culture after preovulatory aging. Our observations provide a first indication that preovulatory aging might affect epigenetic regulation of expression and chromosome constitution in mammalian oocytes. Future studies are needed to obtain a more detailed picture also on other histone modifications and histone patterns in humans.

In conclusion, the current study provides a major step forward in explaining the mechanisms behind the impaired oocyte competence associated with preovulatory aging. It sheds some light on alterations in gene expression and chromatin conformation that are important for normal chromosome distribution, spindle integrity and developmental competence in the oocyte. Both experimental systems showed disturbances, with dramatic changes in oocyte quality and developmental potential. From the observations in the current study, it seems that protein recruitment of several factors is highly regulated and differentially influenced by preovulatory aging. The current observations are relevant in the context of assisted reproduction, since they show the importance of timing of ovulation and the need to develop appropriate *in vitro* culture and maturation protocols for *in vitro* folliculogenesis.
